# Antiatherosclerotic and Cardioprotective Potential of *Acacia senegal* Seeds in Diet-Induced Atherosclerosis in Rabbits

**DOI:** 10.1155/2014/436848

**Published:** 2014-12-07

**Authors:** Heera Ram, Rameshwar Jatwa, Ashok Purohit

**Affiliations:** ^1^Department of Zoology, Jai Narain Vyas University, Jodhpur 342001, India; ^2^Molecular Medicine and Toxicology Lab, School of Life Sciences, Devi Ahilya University, Indore, Madhya Pradesh 452001, India

## Abstract

*Acacia senegal* L. (Fabaceae) seeds are essential ingredient of “Pachkutta,” a specific Rajasthani traditional food. The present study explored antiatherosclerotic and cardioprotective potential of *Acacia senegal* seed extract, if any, in hypercholesterolemic diet-induced atherosclerosis in rabbits. Atherosclerosis in rabbits was induced by feeding normal diet supplemented with oral administration of cholesterol (500 mg/kg body weight/day mixed with coconut oil) for 15 days. Circulating total cholesterol (TC), HDL-cholesterol (HDL-C), LDL-cholesterol (LDL-C), triglycerides, and VLDL-cholesterol (VLDL-C) levels; atherogenic index (AI); cardiac lipid peroxidation (LPO); planimetric studies of aortal wall; and histopathological studies of heart, aorta, kidney, and liver were performed. Apart from reduced atherosclerotic plaques in aorta (6.34 ± 0.72) and increased lumen volume (51.65 ± 3.66), administration with ethanolic extract of *Acacia senegal* seeds (500 mg/kg/day, p.o.) for 45 days to atherosclerotic rabbits significantly lowered serum TC, LDL-C, triglyceride, and VLDL-C levels and atherogenic index as compared to control. Atherogenic diet-induced cardiac LPO and histopathological abnormalities in aorta wall, heart, kidney, and liver were reverted to normalcy by *Acacia senegal* seed extract administration. The findings of the present study reveal that *Acacia senegal* seed extract ameliorated diet-induced atherosclerosis and could be considered as lead in the development of novel therapeutics.

## 1. Introduction

Atherosclerosis is a chronic disease characterized by lipid deposition and inflammation in arterial wall [1]. Accumulation of oxidized cholesterol through a cascade of gradual developing processes results in an unstable atherosclerotic plaque that ultimately bursts and gives rise to myocardial infarction [1, 2]. Atherosclerosis is mainly influenced by hypercholesterolemia and dyslipidemia that are developed through various risk factors, that is, hereditary, sedentary lifestyle, diabetes, and high fat diet consumption [1, 3, 4]. A number of pharmacological agents are available in the market to manage dyslipidemia and atherosclerosis; however the drugs are reported to induce adverse drug reactions [5–7]. Interestingly, herbal nutritional supplement has a potential to ameliorate cardiovascular diseases at different steps in their development without any known side effect [5, 7, 8].

The resident population of Western Rajasthan consumes a specific kind of long-established food known as “Pachkutta” and its main components are* Acacia senegal* L. (Fabaceae) seeds, pod of* Prosopis cineraria* L. (Fabaceae), and* Capparis decidua* L. (Capparaceae) fruit. In general, Rajasthani people who incorporate “Pachkutta” and some long-established foods in their diet are found to report almost nil incidences of cardiovascular system related diseases.* Acacia senegal*, commonly known as Gum Arabica, Kumath, and Rfaudraksha, is a drought or arid region tree. Officinal parts of* Acacia senegal* such as seeds, fruits, leaves, gum, and bark are rich in polyphenols, flavonoids, tannins, saponins, and alkaloids [9, 10]. While* Acacia senegal* gum is used for soothing mucous membranes of the intestine and to treat inflamed skin [11], it is also reported to cure bleeding, bronchitis, malaria, diarrhea, gonorrhea, leprosy, typhoid fever, and upper respiratory tract infections besides possessing antiplatelet and antifertility activities [12–14].

The present study was planned to investigate antiatherosclerotic and cardioprotective role of* Acacia senegal* seed extract, if any, using high fat diet-induced atherosclerotic rabbits as working model. Atherosclerotic plaques in aorta; lumen volume; cardiac lipid peroxidation (LPO); circulating total cholesterol (TC), HDL-cholesterol (HDL-C), LDL-cholesterol (LDL-C), triglycerides, and VLDL-cholesterol (VLDL-C) levels; atherogenic index (AI); and organ (heart, aorta, kidney, and liver) weight were considered as key parameters. Planimetric studies of aortal wall and histopathological studies of heart, aorta, kidney, and liver were studied as supporting parameters to correlate with altered conditions.

## 2. Materials and Methods

### 2.1. Chemicals and Plant Material

Standard drug statin (Atorvastatin) was purchased from a registered local medical store. Glacial acetic acid, hydrogen peroxide, diethylene triamine penta acetic acid, sodium dodecyl sulfate, ethylene diamine tetra acetic acid (EDTA), HCl, xylene, stain chemicals, and sulfuric acid were obtained from E. Merck Ltd., Mumbai, India. All other chemicals were of reagent grade and were purchased from Loba Chemie, Mumbai, India.* Acacia senegal* L. seeds were procured from a registered supplier M/S Bharat seed distributors, Jodhpur, Rajasthan, India, and authenticated by taxonomist. Seeds were ground to powder form and extracted in 70% ethanol for 18 h using soxhlet apparatus; 2% of ground powder was recovered in extract. A voucher specimen was deposited in departmental herbarium (HR-2012-AS).

### 2.2. Animals and Induction of Atherosclerosis

Colony bred adult New Zealand white male rabbits, weighing 1.6 ± 0.2 kg, were used as working model. Animals were housed in metallic wire gauge cages, under controlled light (12 h light: dark) and temperature (23 ± 2°C) controlled room with the provision of standard laboratory feed (Hindustan Lever Ltd., Mumbai, India). Food was supplemented with green leafy and seasonal vegetables and water* ad libitum*. Thirty-two healthy rabbits were divided into four groups of eight each. Animals of group 1 receiving the vehicle, distilled water (5 mL/animal/day, p.o.) served as control, while those of groups 2, 3, and 4 received high fat diet (500 mg cholesterol mixed with 5 mL coconut oil/kg, p.o.) for 15 days to induce atherosclerosis [15]. The average consumption of diet was 200 g/rabbit/day. After 15 days animals of groups 1 and 2 received vehicle, distilled water (5 mL/animal/day, p.o.) and those of groups 3 and 4 received* Acacia senegal* seed extract (500 mg/kg/day, p.o.) and Atorvastatin (0.25 mg/kg/day, p.o.), respectively, for 45 days. Drug or vehicle administration was done by gastric intubation method. Institutional Animal Ethics Committee (IAEC) approved the experimental protocols.

### 2.3. Collection of Blood Samples and Planimetric Studies

After completion of 60 days, overnight fasting animals were sacrificed under ether anesthesia. Blood samples were collected by cardiac puncture method and kept in EDTA coated test tubes and normal tubes for biochemical and hematological assessments. Planimetric studies of aorta wall and atherosclerotic plaque were performed by using Camera Lucida and measured layers (intima, media, and adventitia), lumen volume, and atherosclerotic plaque area, as routinely done in our laboratory [15].

### 2.4. Biochemical Estimations in Tissue and Plasma

Plasma fasting glucose concentration was measured by following enzymatic (glucose oxidase/peroxidase) method, as described elsewhere [16, 17]. Cardiac LPO was studied in microsomal fraction by the reaction of thiobarbituric acid with malondialdehyde in acidic condition [6, 17]. While for the estimation of TC and HDL-C spectrophotometric methods of Allain et al. [18] and Finley and coworkers [19], respectively, and for triglycerides standard, protocol [20] was followed. LDL-C, VLDL-C, and atherogenic index (AI) were calculated using Friedewald's formula [17, 21].

### 2.5. Histopathological Investigations

After exsanguinations heart, aorta, liver, and both the kidneys were removed, quickly freed from blood clots, and washed thoroughly with phosphate buffered saline (0.1 M, pH 7.4) and kept in Bouin's fixatives. Fixed sections were stained with hematoxylin-eosin after passing through ethanol series and observed under Microscope to study hypercholesterolemic diet-induced alterations in histoarchitecture [15, 22].

### 2.6. Statistical Analyses

Values of biochemical assessment, organs weights, and planimetric studies were expressed as mean ± standard error of mean (S.E.M.) and analyzed for ANOVA and* post hoc* Dunnett's *t*-test using SPSS 17 trial version for windows.

## 3. Results

### 3.1. Plasma Lipid Profile and Cardiac LPO

**Figure 1:**
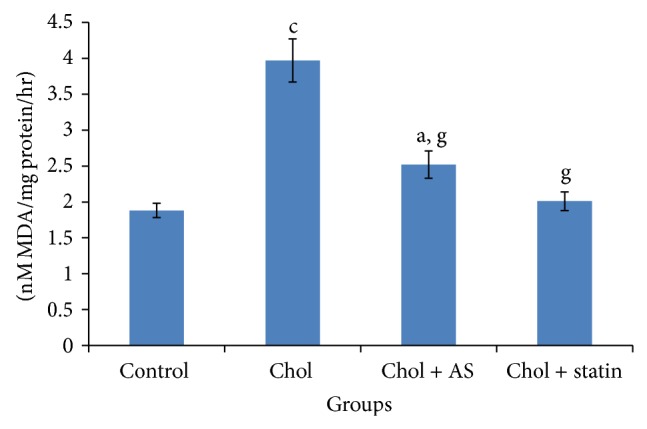
Effects of* Acacia senegal* seed extract (AS, 500 mg/kg/day, p.o.) or statin (0.25 mg/kg/day, p.o.) treatment for 45 days on the alteration in cardiac lipid peroxidation (nM MDA/mg protein/h) in hypercholesterolemic (Chol) diet fed atherosclerotic male rabbits. Data are means ± S.E.M. (*n* = 8); a, *P* ≤ 0.05 and c, *P* ≤ 0.001 as compared to the respective control values and g, *P* ≤ 0.001 as compared to the respective values of the Chol diet fed group.

**Figure 2:**
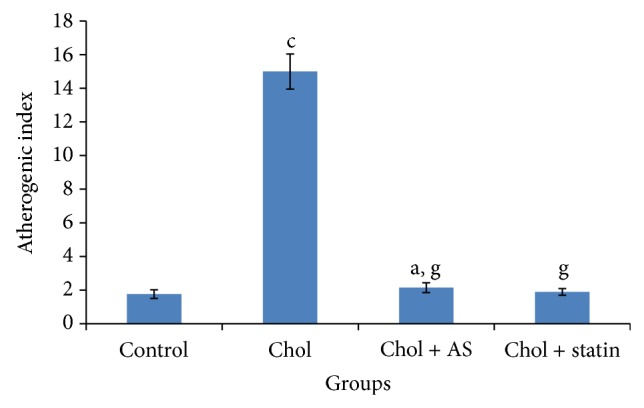
Effects of* Acacia senegal* seed extract (AS, 500 mg/kg/day, p.o.) or statin (0.25 mg/kg/day, p.o.) treatment for 45 days on atherogenic index in hypercholesterolemic (Chol) diet fed atherosclerotic male rabbits. Data are means ± S.E.M. (*n* = 8); a, *P* ≤ 0.05 and c, *P* ≤ 0.001 as compared to the respective control values and g, *P* ≤ 0.001 as compared to the respective values of the Chol diet fed group.

Administration of atherosclerotic diet to rabbits for 15 days increased cardiac LPO, AI value, and circulating TC, LDL-C, VLDL-C, and triglyceride levels (Table 1; Figure 1).* Acacia senegal* seed extract administration for 45 days to atherosclerotic animals reduced TC, triglycerides, LDL-C, VLDL-C, and atherogenic index and cardiac LPO (Table 1; Figure 2).

### 3.2. Planimetric Studies, Histological Observations, and Organs Weight

**Figure 3:**
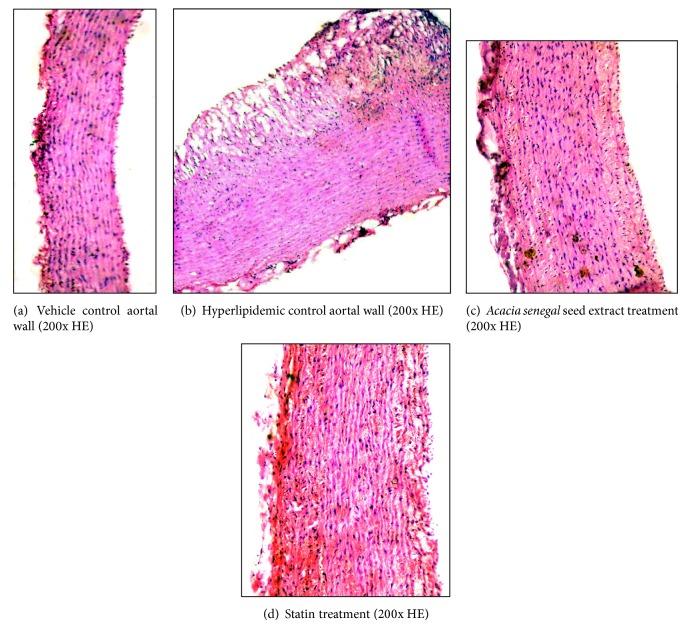
Effects of* Acacia senegal* seed extract (AS, 500 mg/kg/day, p.o.) or statin (0.25 mg/kg/day, p.o.) treatment for 45 days on the alteration in aorta wall in hypercholesterolemic (Chol) diet fed male rabbits (200x H&E).

In vehicle treated control group, aorta wall consisted of three layers, that is, intima, media, and adventitia as normal histoarchitecture. Administration of high fat diet along with cholesterol to rabbits caused a bulging structure of atheromatous plaque at intimal surface (Figure 3).* Acacia senegal* seed extract and statin administration to atherosclerotic rabbits reduced atheromatous plaques at different degrees and increased lumen volume (Table 2). Atheromatous plaque development and fat deposition caused abnormal histoarchitecture of aortal wall in hypercholesterolemic group (Figure 3). On one hand treatment with* Acacia senegal* extract reduced plaque area up to 82.18%, intima area 18.19%, and total wall 28.84% and on the other hand media area and lumen volume were increased to 40.85% and 41.39%, respectively (Table 2). Nonsignificant alterations in heart and kidney weight were observed after hypercholesterolemic diet feeding as well as* Acacia senegal* seed extract and statin administration, except aorta and liver (Table 3).

## 4. Discussion

Findings of the present study reveal that hypercholesterolemic diet fed rabbits not only decreased aorta and lumen area, but also developed dyslipidemia and atherosclerosis. Hypercholesterolmic diet-induced dyslipidemia is quite expected as the diet itself is composed of cholesterol and lipid. Dietary factors play an important role in the development of various diseases, including that of cardiovascular diseases. Landmark epidemiological studies reflect that diets rich in fruits, herbs, and spices are associated with a low risk of cardiovascular diseases [8, 23, 24]. Mammals are sensitive to atherosclerosis, induced by dietary cholesterol because they are unable to increase sterol excretion, resulting into enhanced liver export of cholesteryl ester-rich lipoproteins into the circulation [4, 25, 26].

In the present study, high fat diet-induced hypercholesterolemia and atherosclerosis are an outcome of accumulation of cholesterol by various mechanisms, as reported earlier [5, 27]. However, treatment with* Acacia senegal* seed extract reduced total cholesterol, LDL-cholesterol, and VLDL-cholesterol as well as AI, reflecting the potential of plant extract in amelioration of diet-induced atherosclerosis and cardiac toxicity. However, hypolipidemic and antiatherosclerotic activities of* Acacia senegal* seed extract might be an outcome of its active ingredients [9, 28]. Indeed, histoprotective nature of* Acacia senegal* seed extract was further supported by the observations made on the volumes of total wall area, lumen, intima, media, adventitia, and plaque.

In fact, plant derived active principles, namely, polyphenols, tannins, flavonoids, polyphenols, alkaloids, and so forth, are reported to reduce cholesterol production in liver and to inhibit the activities of cholesterol biosynthetic enzymes [23, 29]. This fact was further supported by histological examinations made on the tissues of* Acacia senegal* seed extract treated aorta, revealing restoration of aortal walls and volume to normalcy and absence of plaques. However, antiatherosclerotic and hypolipidemic effects might be an outcome of depletions of deposited fatty contents and reduced peroxidation of lipid contents following* Acacia senegal* seed extract, as suggested by earlier workers [5, 30–32]. Interestingly, following* Acacia senegal* seed extract administration, aorta and liver weights were also brought to normal. Indeed, plant derived active principles such as polyphenols, saponins, alkaloids, and flavonoids are reported to suppress LDL-receptors and promote lipolytic activities [4, 8]. While restoration of tissue histology following plant extract administration might be an outcome of inhibitions in the activities of HMG CoA reductase enzyme which catalyzes conversion of HMG CoA to mevalonate, a rate limiting step in the formation of endogenous cholesterol leading to the decrease in the intracellular status of cholesterol [25, 33], it is speculated that* Acacia senegal* seed might lower LDL-cholesterol through inhibiting hepatic cholesterol biosynthesis and VLDL-cholesterol production which is the source of LDL-cholesterol production. This fact is further supported by the findings made on lipid profile, which was reduced following plant extract administration. These alterations also followed suppression of VLDL particle maturation and secretion from liver, decrease the VLDL level in plasma, and further decrease the LDL level and AI value [31].

Atherosclerotic diet fed animals also exhibited damage in cardiac tissues as evidenced by increase in LPO. The enhanced tissue LPO along with altered cardiac and aorta histology and dyslipidemia manifests the toxic nature of the hypercholesterolemic diet in different tissues, as suggested earlier by other workers [4, 27], while* Acacia senegal* extract administration resulted in amelioration of oxidative stress evidenced by the reduced LPO and restoration of cardiac histology to normalcy as well as lipid profiles. In fact, following plant extract treatment reversal of altered cardiac histopathology and LPO might be an outcome of its active principles, as plant extracts are reported to reduce oxidative stress [14, 34–36].

In conclusion, the findings of the present study provide justification for inclusion of* Acacia senegal* seeds in traditional Rajasthani diet “Pachkutta” as the extract ameliorated hypercholesterolemic diet-induced atherosclerosis without any adverse effects and could be considered as lead in the development of novel therapeutics. However, studies on the isolated active principles are required to recommend the seeds as therapeutics for cardiovascular diseases.

## Figures and Tables

**Table 1:** Effects of *Acacia senegal* seed extract (AS, 500 mg/kg/day, p.o.) or statin (0.25 mg/kg/day, p.o.) treatment for 45 days on the alteration in plasma concentration of total cholesterol (TC), triglyceride (TG), high-density lipoprotein cholesterol (HDL-C), low-density lipoprotein cholesterol (LDL-C), and very low-density lipoprotein cholesterol (VLDL-C), all in mg/dL and on TC/HDL-C ratio and on atherogenic index in hypercholesterolemic (Chol) diet fed atherosclerotic male rabbits.

Groups	TC	TG	HDL-C	LDL-C	VLDL	CHO/HDL
Control	89.48 ± 6.25	101.40 ± 8.23	32.31 ± 1.3	37.60 ± 5.1	20.40 ± 1.2	2.98 ± 0.02
Chol	523.59 ± 33.66^c^	366.72 ± 15.23^c^	32.31 ± 1.2^d^	419.41 ± 6.50^c^	73.37 ± 2.38^c^	17.47 ± 0.69^c^
Chol + AS	92.50 ± 6.43^d,g^	148 ± 12.80^a,g^	29.50 ± 2.11^d,h^	35.05 ± 1.39^d,g^	28.50 ± 2.36^b,g^	3.12 ± 0.22^a,g^
Chol + statin	84.49 ± 4.79^d,g^	95.86 ± 07.72^d,g^	29.79 ± 1.58^d,h^	35.67 ± 4.43^d,g^	20.85 ± 2.33^d,g^	2.81 ± 0.14^a,g^

Data are means ± S.E.M. (*n* = 8); ^a^ *P* ≤ 0.05; ^b^ *P* ≤ 0.01; ^c^ *P* ≤ 0.001; and ^d^nonsignificant as compared to the respective control values. ^g^ *P* ≤ 0.001 and ^h^nonsignificant as compared to the respective values of the Chol diet fed group.

**Table 2:** Effects of *Acacia senegal* seed extract (AS, 500 mg/kg/day, p.o.) or statin (0.25 mg/kg/day, p.o.) treatment for 45 days on the alteration in total wall area (TWA), lumen, intima, media, adventitia, and plaque, all in mm (images of Camera Lucida at 4 × 8 magnification; all in % of total area) in hypercholesterolemic (Chol) diet fed atherosclerotic male rabbits.

Groups	TWA	Lumen	Intima	Plaque	Media	Adventitia
Control	48.42 ± 1.99	49.74 ± 1.44	9.62 ± 0.19	0.0	27.0 ± 0.80	10.34 ± 0.02
Chol	67.16 ± 4.61^a^	30.27 ± 2.52^a^	10.06 ± 0.49^d^	35.59 ± 1.01^c^	14.23 ± 0.66^c^	10.91 ± 0.13^a^
Chol + AS	47.79 ± 3.67^d,e^	51.65 ± 3.66^a,g^	8.23 ± 0.78^d,e^	6.34 ± 0.72^c,g^	24.06 ± 0.89^d,g^	10.06 ± 0.67^a,e^
Chol + statin	49.69 ± 1.02^d,e^	48.93 ± 2.44^d,e^	9.98 ± 0.39^d,h^	0.0	27.01 ± 1.84^d,g^	10.39 ± 0.34^d,e^

Data are means ± S.E.M. (*n* = 8); ^a^ *P* ≤ 0.05; ^b^ *P* ≤ 0.01; ^c^ *P* ≤ 0.001; and ^d^nonsignificant as compared to the respective control values. ^e^ *P* ≤ 0.05; ^g^ *P* ≤ 0.001; and ^h^nonsignificant as compared to the respective values of the Chol diet fed group.

**Table 3:** Effects of *Acacia senegal* seed extract (AS, 500 mg/kg/day, p.o.) or statin (0.25 mg/kg/day, p.o.) treatment for 45 days on the alteration in liver, heart, kidney, and aorta weight, all in gm/Kg body weight in hypercholesterolemic (Chol) diet fed atherosclerotic male rabbits.

Groups	Liver	Heart	Kidney	Aorta
Control	26.34 ± 1.35	2.12 ± 0.18	6.74 ± 0.41	0.16 ± 0.05
Chol	40.01 ± 1.35^c^	2.85 ± 0.12^d^	7.23 ± 0.48^d^	0.41 ± 0.17^c^
Chol + AS	24.65 ± 1.51^d,g^	2.12 ± 0.17^d,h^	6.29 ± 0.43^d,h^	0.25 ± 0.13^c,g^
Chol + statin	25.76 ± 1.32^d,g^	2.57 ± 0.14^d,h^	6.67 ± 0.32^d,h^	0.27 ± 0.12^c,g^

Data are means ± S.E.M. (*n* = 8); ^c^ *P* ≤ 0.001; ^d^nonsignificant as compared to the respective control values; ^g^ *P* ≤ 0.001; and ^h^nonsignificant as compared to the respective values of the Chol diet fed group.
